# Preclinical Models of Donation-After-Circulatory-Death and Brain-Death: Advances in Kidney Preservation and Transplantation

**DOI:** 10.3390/biology14101415

**Published:** 2025-10-14

**Authors:** Tamara S. Ortas, Omer Choudhary, George J. Dugbartey, Alp Sener

**Affiliations:** 1 Department of Microbiology & Immunology, Western University, London, ON N6A 5C1, Canada; 2 Matthew Mailing Center for Translational Transplant Studies, London Health Sciences Center, Western University, London, ON N6A 5A5, Canada; 3 Department of Physiology & Pharmacology, Western University, London, ON N6A 5C1, Canada; 4 Multi-Organ Transplant Program, London Health Sciences Center, Western University, London, ON N6A 5A5, Canada; 5 Department of Surgery, Division of Urology, London Health Sciences Center, Western University, London, ON N6A 5A5, Canada; 6 Department of Physiology & Pharmacology, Accra College of Medicine, Accra P.O. Box CT9828, Ghana

**Keywords:** kidney transplantation, donation-after-brain-death (DBD), donation-after-circulatory-death (DCD), ischemia–reperfusion injury (IRI), static cold storage (SCS), hypothermic machine perfusion (HMP)

## Abstract

**Simple Summary:**

Kidney transplantation is the optimal treatment for patients with kidney failure. Unfortunately, the demand for donor kidneys outweighs the number of donor kidneys available. This has created a global donor kidney shortage crisis and a long waiting list of transplant recipients, and thus, has necessitated the use of kidneys from deceased donors. However, compared to living donor kidneys, kidneys from deceased donors are more vulnerable to injury due to the lack of blood supply upon death, and injury from the transplantation procedure involving cold preservation and restoration of blood supply upon transplantation. In recent years, alternative preservation methods, including addition of protective agents to storage solutions, have emerged, with the goal of improving the quality and function of kidneys from deceased donors, reducing post-transplant complications, and eventually reducing the number of patients on the transplant waiting list. In this review, we discussed the mechanisms of injury in deceased donor kidneys. In addition, we also discussed within the limits of present literature the preservation strategies tested in animal models. Finally, the review ends with a discussion on emerging therapeutic interventions aimed at improving adverse post-transplant outcomes.

**Abstract:**

Chronic kidney disease (CKD) affects over 10% of the global population, with end-stage renal disease (ESRD) necessitating renal replacement therapy. Kidney transplantation remains the optimal treatment for ESRD. However, the global donor kidney shortage crisis has led to increased reliance on deceased donor kidneys. Donors are classified as either donation after brain death (DBD) or donation after circulatory death (DCD), each associated with distinct ischemic injuries that impact graft function. Ischemia–reperfusion injury (IRI) plays a pivotal role in transplant outcomes, triggering oxidative stress, inflammation, and endothelial dysfunction. While static cold storage (SCS) remains the gold standard for organ preservation, alternative strategies such as hypothermic or normothermic machine perfusion (HMP and NMP), use of oxygen carriers during storage, and supplemental compounds to storage solutions have emerged, offering potential benefits in preserving graft viability. This review explores the cellular and molecular mechanisms of ischemic injury in deceased donor kidneys, preservation strategies tested in preclinical models, and emerging therapeutic interventions aimed at improving adverse post-transplant outcomes.

## 1. Introduction: The Need for Renal Grafts from Deceased Donors

Chronic kidney disease (CKD) is a progressively worsening condition that has emerged as a leading cause of morbidity and mortality globally over recent decades [1]. It impacts over 10% of the global population, equivalent to approximately 800 million individuals [1]. CKD is identified by the irreversible decline in kidney function, categorized into five stages based on the severity of renal impairment. The final and most severe stage of CKD, known as end-stage renal disease (ESRD), manifests when less than 15% of normal kidney function remains, necessitating renal replacement therapy (RRT). The predominant form of RRT for individuals with ESRD is hemodialysis (HD), a process where an external machine temporarily substitutes for the kidneys [2]. Although HD prevents imminent death, it is associated with significant challenges for both patients and healthcare systems, including potential complications such as hypotension, the requirement for frequent treatment sessions, risk of infections, and substantial costs exceeding USD 90,000 annually per patient, and a modest five-year survival rate of 40–55% [3].

Currently, kidney transplantation stands as the optimal RRT for patients with ESRD, boasting an impressive five-year survival rate of up to 90% when the renal graft is from a living donor, and sparing patients many of the complications associated with HD. Nonetheless, the scarcity of renal grafts from living donors relative to the high demand worldwide poses a significant global donor kidney shortage crisis. Consequently, renal grafts from deceased donors have become a common source for transplantation, offering a slightly lower, yet still favorable, five-year survival rate of 80% [4]. Traditionally, organ grafts were obtained from donors who experienced circulatory death. However, since the 1960s, the adoption of brain death criteria—defined as the complete and irreversible cessation of brain stem function without the stoppage of heart function—has allowed for the use of organs from donors after brain death (DBD), which now constitutes the majority of transplanted organs due to their superior outcomes compared to those from donors after circulatory death (DCD) [5]. Although several review articles have discussed aspects of DCD and DBD transplantation, the majority either focus on clinical outcomes in isolation or provide broad overviews of preservation strategies without delving into the experimental foundations. What is notably absent in the literature is a comprehensive synthesis that systematically examines animal models of both DCD and DBD, as well as the lack of how these preclinical studies have shaped our understanding of ischemia–reperfusion injury (IRI) and preservation, and critical evaluation of their translation into the clinical setting. By bridging mechanistic insights from experimental models with their implications for clinical practice, this review fills an important gap, offering a framework that links basic science discoveries to ongoing and future advances in transplantation.

## 2. Cellular and Molecular Mechanisms of Damage in Deceased Donor Kidneys

Renal grafts from DCD or DBD experience ischemia, during which they do not receive a blood supply. Compared to renal grafts from living donors, kidneys from deceased donors experience significantly longer ischemic time, which negatively impacts graft quality and function, and increases the risk of post-transplant complications [6]. Due to hypoxia during ischemia, cells of ischemic tissues are unable to produce adequate energy to sustain cellular processes [7]. At the cellular level, this results in a switch from efficient aerobic metabolism to less efficient anaerobic metabolism, leading to a significant drop in the production of the energy molecule, adenosine triphosphate (ATP). Activation of the anaerobic metabolism causes acidosis due to lactic acid accumulation, lowering intracellular pH [7,8]. This acidosis destabilizes lysosomal membranes, causing enzymes to leak into the cytoplasm. Decreased ATP production also impairs functioning of ion pumps such as Na^+^/K^+^ transporter and the Ca^2+^ pump, leading to increased levels of intracellular succinate and calcium, and causing cellular edema [6,8,9].

Although restoration of blood supply (reperfusion) is crucial for rescuing ischemic tissues, paradoxically, reperfusion of the ischemic graft during transplantation exacerbates cellular damage. The collective damage that results from ischemia followed by reperfusion is termed IRI. Reperfusion is associated with an over-production of reactive oxygen species (ROS) [6,8]. This surge depletes intracellular antioxidants such as glutathione (GSH) [10]. As a result, oxidative stress develops and damages cellular structures, enzymes, and DNA. In addition, the interaction of ROS with these cellular structures can also result in formation of toxic metabolites within the cells. For example, ROS can cause lipid peroxidation upon reaction with lipids, resulting in formation of secondary metabolites such as malondialdehyde (MDA) and 4-hydroxynonenal (4-HNE) [11]. MDA and 4-HNE are mutagenic and cytotoxic substances, respectively, and exacerbate cell injury. In addition, intracellular iron ions undergo the Fenton reaction with ROS to form more reactive ROS [10], whose interactions with membrane phospholipids lead to loss of cellular membrane integrity. This form of cell death is termed “*ferroptosis*”, where iron ions modulate cell death by promoting lipid peroxidation. Under physiological conditions, the enzyme glutathione peroxidase 4 (GPX4) reduces the lipid peroxides to nontoxic lipid alcohols to prevent ferroptosis. However, GPX4 requires the cofactor GSH for its activity, which is rapidly depleted in the cells that experienced IRI.

**Figure 1:**
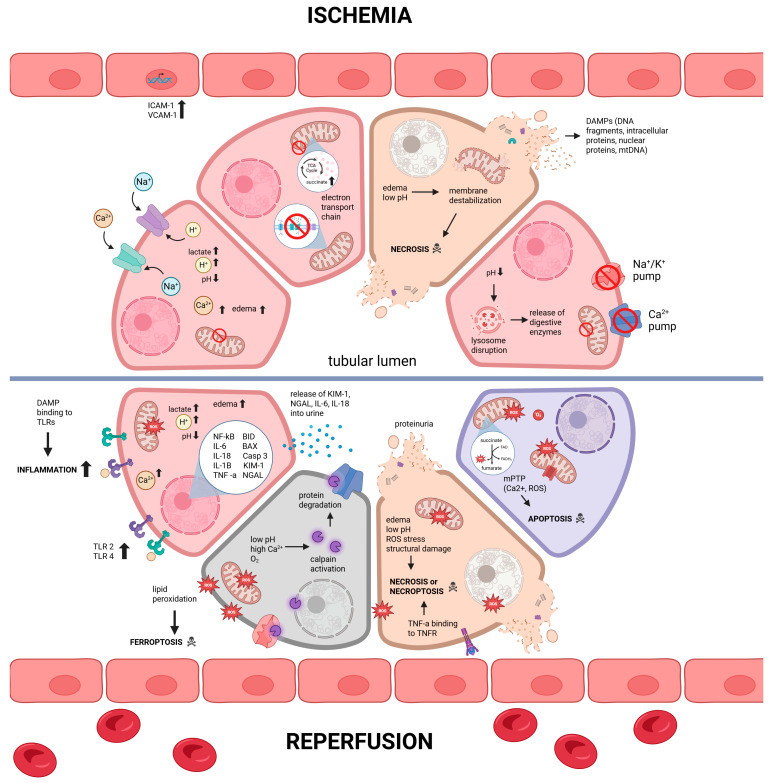
Molecular pathways underlying renal graft damage during ischemia–reperfusion injury. The top panel depicts cellular and molecular changes that occur during ischemia, while the bottom panel illustrates the subsequent pathological changes during reperfusion. Abbreviations: danger-associated molecular patterns (DAMPs), mitochondrial permeability transition pore (mPTP), reactive oxygen species (ROS), and tumor necrosis factor receptor (TNFR).

Besides ferroptosis, ROS can also trigger other pathways within the cells that result in cell death. ROS that are generated during reperfusion, in combination with high intracellular Ca^2+^ concentrations, can trigger the opening of mitochondrial permeability transition pore (mPTP), which ultimately results in oxidative stress-mediated necrosis or apoptosis [12,13]. Although there are multiple sources of ROS production during reperfusion, a major source of ROS generation is due to rapid oxidation of accumulated succinate during reoxygenation of the ischemic tissue [9,14]. Restoration of physiological pH during reoxygenation, together with increased intracellular Ca^2+^, activates calpains. These are calcium-dependent proteases that degrade proteins in the cytoskeleton, endoplasmic reticulum, and mitochondria [6]. Moreover, cytokines such as interleukin 1-alpha (IL-1*α*) released from necrosed cells have been found to stimulate extracellular release of tumor necrosis factor-alpha (TNF-*α*) from sublethally injured kidney epithelial cells [15]. TNF-*α* is a well-characterized inducer of necroptosis, a form of necrosis that occurs in a genetically programmed fashion [16]. Adverse cellular conditions that arise during IRI, including ATP depletion, ROS generation, intracellular Ca^2+^ accumulation, cellular edema, and lysosomal membrane disruption, may also trigger the necroptotic pathway [17]. Figure 1 summarizes and illustrates the molecular changes and pathways of damage during IRI in the renal graft.

Furthermore, endothelial cells in blood vessels within the renal graft are also affected in IRI. The shear stress that is induced by the blood flow ceases during ischemia. This shear stress is important for the endothelium, as it stimulates generation of vasoprotective factors such as Krüppel-like Factor 2 (KLF2) [18]. KLF2 is a transcriptional integrator of vasoprotective phenotype through its regulation of anti-inflammatory, pro-inflammatory, and antioxidant genes. KLF2 downregulates expression of pro-inflammatory proteins such as vascular cell adhesion molecule 1 (VCAM-1) and stimulates endothelial nitric oxide synthase (eNOS) activity. In the absence of blood flow, however, KLF2 expression is downregulated [18].

Changes during ischemia can result in failure of the renal microvasculature, preventing blood flow to effectively oxygenate the renal tissue even when the renal graft is transplanted [19]. This phenomenon, referred to as *“no-reflow phenomenon”,* can result in prolongation of the ischemic period. No-reflow is caused by loss of endothelial cells and capillary occlusion within the kidney. Capillary occlusion is thought to be a result of swelling of the endothelial cells due to edema and adhesion of leukocytes to the endothelium [20]. Adhesion of leukocytes such as neutrophils to the endothelium can further contribute to endothelial damage through degranulation and release of hydrolytic enzymes [19].

In addition to IRI and microvascular damage, renal grafts provoke a robust inflammatory response from the immune system of the recipient, resulting in further graft damage [21]. As pathological changes that occur due to IRI result in necrosis, the necrosed cells release danger-associated molecular patterns (DAMPs) such as ATP, fragments of DNA, IL-1*α*, and high mobility group box 1 (HMGB1) to the extracellular environment, which interact with pattern recognition receptors (PRRs) of immune cells as well as epithelial and endothelial cells of the kidney [22,23]. Some of the PRRs that are found on renal epithelial cells are toll-like receptors (TLRs) such as TLR2 and TLR4, which interact with HMGB1 and result in nuclear translocation of nuclear factor kappa B (NF-кB), an inflammation-related nuclear factor [24]. Nuclear translocation of NF-кB results in activation of pro-inflammatory pathways, including NLRP3 inflammasome [25]. Activation of the NLRP3 inflammasome causes cleavage of its effector domain pro-caspase 1 into caspase-1. Activity of caspase-1 results in cleavage and activation of the pro-inflammatory cytokines, IL-1β and IL-18, thereby contributing to recruitment of immune cells. Also, activity of IL-1β can stimulate increased expression of adhesive molecules on mesenchymal and endothelial cells, and IL-18 upregulates production of interferon gamma (IFN-ɣ) in natural killer (NK) cells. Increased expression of adhesive proteins on cell surfaces is observed after one hour of IRI, and it is associated with more infiltration of neutrophils [26].

Loss of barrier function of endothelial cells in the renal microvasculature is another pathological change that may result in increased leukocyte infiltration in the donor kidney due to IRI-induced endothelial damage, and leads to increased transmigration of leukocytes such as macrophages, neutrophils, and NK cells [22]. Once activated, macrophages release pro-inflammatory cytokines such as IL-6 and TNF-*α*, which exacerbate renal IRI through upregulation of TLR2 and TLR4 expression on the renal epithelial cells [27], and together with other pathological pathways, can result in both short- and long-term post-transplant complications such as delayed graft function (DGF), slow graft function, and primary non-function [28,29,30].

### 2.1. Renal Grafts from Donation-After-Cardiac-Death

A DCD graft is one where cardiac function was ceased in the donor prior to procurement of the graft [4]. Upon cardiac arrest, the heart no longer supplies blood to the kidneys, resulting in ischemia. The period between cardiac arrest and organ procurement is termed warm ischemic time (WIT), as the kidney is in hypoxic conditions at a physiological temperature. Ischemia at physiological conditions is particularly damaging to the kidneys, as metabolic demand of the kidney is high due to high mitochondrial density in renal cells. Due to the adverse effects of WIT, DGF occurs more commonly in DCD renal grafts. In fact, about 30–50% of all DCD grafts result in DGF [31]. DCD grafts that experience prolonged periods of WIT are particularly susceptible to DGF after transplantation [32]. The occurrence of DGF requires increased healthcare costs for transplant recipients, as it requires a return to HD, biopsy, acute rejection, decreased long-term survival of the renal graft, and another transplant, all of which prolong hospital stay [28,33].

DCD grafts are categorized into different types based on timing and circumstances of death and procurement. The most commonly used categorization system is the Maastricht classification system [34,35], which is briefly explained in Table 1. The DCD grafts that are most commonly transplanted are from Category III, as the grafts from this category are procured in a controlled setting, where WIT is known with certainty and is minimized by the medical team.

### 2.2. Renal Grafts from Donation-After-Brain-Death

Transplantation of DBD kidneys started in the 1950s and 1960s when the concept of brain death emerged, but most notably was highlighted with the publication by Harard’s medical school in 1968, “*A Definition of Irreversible Coma.*” This report brought out the criteria for brain death, which was eventually accepted as a legal death in many countries, including the United States of America, Canada, and Germany [36]. However, across many different cultures and countries, DBD is not accepted. For example, China does not have any legal recognition of brain death [36]. However, a new concept, referred to as donation after brain death followed by cardiac death (DBCD), has been established in China for organ transplantation [36]. DBCD is a hybrid between DBD and DCD. It acknowledges brain death as part of the criteria but waits for cardiac death to occur before the organ is procured [36]. This classification system proposes many solutions, especially in areas where cultural views do not support DBD grafts. On one hand, it leads to an increased organ donation rate as noted by the authors of the study [36]. In addition, it also leads to similar outcomes as DBD, as it involves a shorter WIT during procurement [36]. Most notably, the study showed that a one-year graft survival and DGF of DBCD kidneys were better than both DBD and DCD [36]. Nevertheless, the acceptance of brain death as a criterion for legal death is a stepping stone to allow for the development of legal and ethical frameworks for organ donation from these donors. As an example, the Uniform Anatomical Gift Act, established in the United States, standardized the process [37]. With this, organ transplantation, including the kidneys, has been performed using DBD grafts.

A DBD graft is obtained from donors who have experienced brain death while on life support. In this case, the donor’s heart is still functioning and continues to provide blood to the organs, including the kidneys [38]. DBD involves the death of the brain stem, which leads to significant hemodynamic instability along with metabolic and hormonal changes [39]. It has been reported that brain death leads to changes in liver response, noted by increased aerobic glycolytic activity, with a possible link to catecholamine storm [39]. The catecholamine storm leads to increased energy demands, which are fulfilled with an upregulated aerobic glycolytic activity [39]. However, when looking at the kidney’s response to brain death, contrasting trends were observed. There was a metabolic shift toward anabolic glycolysis coupled with ATP depletion [39]. In addition, increased oxidative stress along with decreased renal perfusion were noted, which required antioxidant and vasodilation for a better transplant outcome [39].

**Figure 2:**
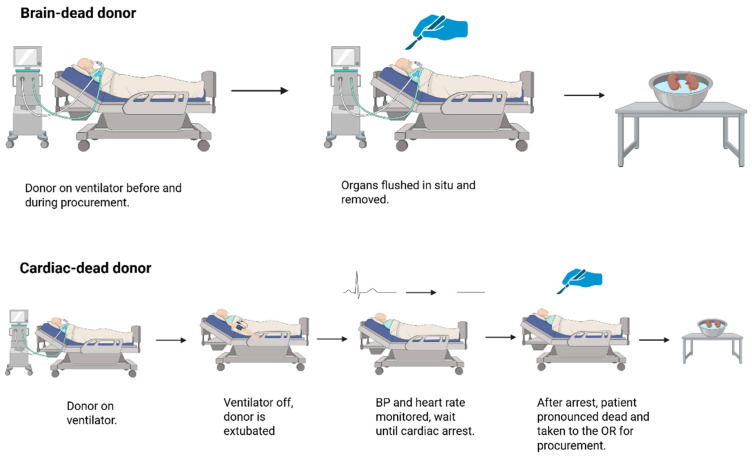
Procurement of brain-dead donor (DBD) and cardiac-dead donor (DCD) organs. Brain-dead donors remain on ventilators throughout the procurement process, allowing continuous oxygenation of organs until they are flushed and placed in cold storage. In contrast, cardiac-dead donors are withdrawn from ventilatory support, and the surgical team waits for cardiac arrest to confirm death before beginning procurement. The absence of blood circulation following cardiac arrest marks the onset of warm ischemia in DCD organs, which transitions to cold ischemia once the organs are flushed and stored.

Unlike DCD renal grafts, DBD grafts do not experience extensive ischemic periods under physiological temperatures [38] (Figure 2). Studies have shown that this difference accounts for the reduced incidence of delayed graft function (DGF) for DBD grafts [36,40]. Additionally, DCD kidneys are discarded at a higher rate compared to DBD kidneys [41]. In the US, for example, 3997 of 11,482 DCD kidneys that were recovered in 2023 were discarded, corresponding to 34% of all DCD kidneys, whereas 4577 of 19,314 DBD kidneys were discarded, corresponding to 24%. Table 2 summarizes the rate of clinical outcomes of DBD and DCD kidneys [41].

The duration of brain death (BDur) has also been shown to impact renal graft function and transplant outcomes, with a longer BDur being associated with lower DGF and improved adverse post-transplant outcomes [42]. In addition, graft survival 1 and 3 years post-transplantation was better for those organs with a longer BDur [42]. While the mechanisms of this association were not studied, the authors hypothesized that it could be due to a prolonged period of appropriate donor resuscitation and appropriate management in the intensive care unit. In the same study, kidneys from donors younger than 55 years decreased the odds of DGF for every additional hour of BDur [42].

Generally, DBD grafts have much better post-transplant outcomes compared to DCD grafts, as DCD grafts are more susceptible to cold ischemic injury and have higher odds of DGF [43]. These outcomes were assessed on various markers such as estimated glomerular filtration rate (eGFR) and graft survival.

### 2.3. Animal Models of Donation-After-Brain-Death

Animal models have played a significant role in examining the physiological and chemical changes in DBD. To date, different animals have been used, and various procedures have been implemented to successfully create a DBD model. One study established a porcine model of DBD through the use of a balloon catheter, which, upon inflation, increased intracranial pressure and led to subsequent brain death [44]. Brain death was confirmed through various signs, including diabetes insipidus, tachycardia, and hypertensive/hypotensive episodes [44]. Other animal models involving rats and dogs have also been studied extensively. For example, another study followed a similar method on rats to induce brain death [45]. They inflated a balloon catheter and confirmed brain death via flatline EEG, diabetes insipidus, absence of brainstem reflexes, and blood pressure fluctuations [45].

While animal models of DBD have been invaluable for understanding the physiological and biochemical consequences of brain death, they also present important limitations. First, the induction of brain death in animal models is often achieved through artificial methods such as balloon catheter inflation, which do not fully replicate the heterogeneous causes of brain death in humans (e.g., traumatic brain injury, stroke, anoxia). Second, the progression of brain death in animals tends to occur rapidly, whereas in human donors, brain death is often a more gradual process accompanied by longer periods of hemodynamic instability and clinical interventions. Third, the smaller size and different physiology of rodents, and to some extent dogs, may limit the direct translatability of findings to human DBD donors, particularly with respect to cardiovascular responses, hormonal fluctuations, and immunological changes [45]. Finally, most animal models are performed under controlled experimental conditions without the confounding comorbidities (such as hypertension, diabetes, or atherosclerosis) that are common in human donor populations. These differences may restrict the applicability of findings from DBD animal studies to the clinical transplant setting.

## 3. Animal Models of Preservation of Renal Grafts from Deceased Donors

Deceased donor organs are particularly susceptible to IRI, as they typically undergo a prolonged period of ischemic storage prior to transplantation. An active area of research in the field of organ transplantation involves studying graft preservation strategies that would allow for optimal preservation of organs from deceased donors during ischemic storage to minimize tissue damage, improve graft quality and function, and reduce post-transplant complications. Animal models are actively used to study efficiency of techniques in order to refine and optimize renal graft preservation.

Initial studies using animal models to investigate kidney transplants focused on determining the maximum duration after death within which the organ remains suitable for transplantation [46]. Research from canine models of isotransplantation showed that if the kidney experiences ischemia for less than an hour while in the donor’s body, it is likely to function well post-transplant, albeit with temporary complications. However, if the ischemic period extends to 2 h, the kidney is expected to suffer some damage, particularly tubular necrosis. Beyond 3 h, the likelihood of the kidney functioning adequately in the recipient is significantly reduced [46]. A recent murine study that evaluated the impact of every additional minute of warm ischemia on donor kidneys revealed that every minute of warm ischemia contributes to renal damage, and 26 min or longer of warm ischemia results in severe renal injury [47]. A recent DCD porcine study that subjected the kidney to warm ischemia prior to 8 h of cold storage and autotransplantation revealed that all donor kidneys that underwent 120 min of warm ischemia prior to cold storage suffered significant damage, which was associated with DGF in patients [28].

### 3.1. Historical Account of Static Cold Storage of Renal Grafts

After cardiac arrest, kidneys experience ischemia in situ at physiological temperatures, which is a main source of damage to DCD grafts. At these temperatures, the metabolic rate and oxygen demand of the cells are high, accelerating their damage [46]. Temperatures below 10 °C can decrease the metabolic demand of the kidneys by 90%, prompting renal graft preservation at such temperatures [48]. Some of the earliest animal models that evaluated effects of temperature on ischemic organs involved cooling of the organ in situ by surrounding the organ with ice packs after inducing ischemia [46]. Cooling the kidney in situ to temperatures of 10 °C and 20 °C during a 6-h period of ischemia resulted in survival rates of 75% and 30% in dogs, respectively. However, 100% mortality was recorded in control dogs when the kidneys were not cooled [46]. To translate this finding clinically, ex vivo storage of organs in cold temperatures before transplantation was evaluated through animal models. An experiment from 1963 evaluated the length of time that renal grafts can be preserved at 4 °C. This experiment showed that canine kidneys can withstand up to 12 h of ischemia when they are kept in Hank’s solution, which mimics the extracellular fluid at 4 °C, and can function immediately after transplantation, maintaining normal levels of creatinine in the bloodstream [46]. However, renal graft preservation for 24 h was unsuccessful due to injury from prolonged cold ischemic time [46]. Nonetheless, early work on subjecting ischemic donor kidneys from animals to hypothermia prior to their transplantation laid the foundation for the current gold standard of organ preservation, which is static cold storage (SCS).

### 3.2. Renal Graft Preservation Time: Lessons from Animal Models

SCS of renal grafts at 0–4 °C for up to 12 h on ice has shown promising results in several studies. Some of the early animal studies that focused on extending the preservation time of organ grafts relied on perfusing the kidney with a solution that resembled the intracellular fluid of the kidneys. In a canine model, storage of donor kidneys in saline at 0–4 °C resisted ischemia for up to 16 h [49]. This experiment also evaluated the effects of “Solution C”, which was a solution designed to simulate the intracellular environment of the kidney, containing high potassium and low sodium concentrations, with the intention of ameliorating edema that results from ischemic conditions by surrounding renal cells with a solution that resembles their internal fluid compositions. Solution C-treated renal grafts had lower serum creatinine after exposure to up to 30 h of cold ischemia [49]. The Solution C set the foundation for designing storage solutions for transplant organs, and was referred to as the “Collins solution”. Although this solution was effective for kidneys that were placed immediately in cold storage following warm ischemia, it was shown to be ineffective for donor kidneys that were exposed to up to 20 min of warm ischemia prior to beginning of storage, which is the case for many DCD organs [50]. The Collins solution was later modified to “Euro-Collins Solution (EC solution)” through addition of a higher concentration of glucose (195 mmol instead of 140 mmol) and removal of magnesium, which was shown to effectively preserve renal grafts for up to 24 h in SCS in a DCD canine model in which the renal grafts were subjected to 35 min of warm ischemia [51]. Hence, renal graft preservation in EC solution became the clinical practice in Europe in the early 1980s [51]. Later, a more improved graft storage solution, known as the University of Wisconsin (UW) solution, was formulated by Belzer and his team [52,53]. In a comparative canine model of kidney transplantation, preservation of renal grafts in UW solution at 4 °C for 72 h resulted in significantly higher graft viability after transplantation compared to renal grafts stored in EC solution [53,54]. Table 3 summarizes the studies discussed in this section with the use of various storage solutions.

### 3.3. Machine Perfusion as an Alternative to Static Cold Storage

The earliest preclinical models of renal graft preservation in the early 20th century were based on perfusion of the grafts with autologous blood. These models were not convenient, as they required large amounts of blood from the donor, and the bulky and heavy perfusion equipment of the time was inconvenient for transportation; hence, they were not brought into clinical practice [54]. With the invention of compact modern pumps that occupy less space, and with the invention of oxygenating pumps, clinical feasibility of perfusion storage of renal grafts was evaluated with animal models. For example, preservation of canine renal grafts for 3 days through pulsatile machine perfusion at 8–12 °C was successfully performed in 1967 using a plasma-derived perfusate [55]. In this experiment, lipoproteins were removed from the plasma prior to using it for perfusion of the kidneys, as lipoproteins were found to aggregate at hypothermic temperatures and block renal microvasculature [56]. All the experimental dogs that received autologous kidneys that were preserved for 72 h survived, and their blood urea nitrogen (BUN) returned to pre-nephrectomy levels within 5 weeks after transplantation of the kidney. Moreover, histological examination of the transplanted kidneys after 5 weeks of transplantation showed normal kidney architecture [56]. This observation provided the basis for blood-origin perfusates for renal grafts, and was successfully implemented into clinical practice in 1968 by transplantation of a kidney that was preserved through hypothermic machine perfusion (HMP) with this plasma-derived solution [57]. However, the use of plasma was not preferred, as it subjected the preserved organ graft to possible infections and contamination. Further work involved evaluation of preservation solutions as perfusates for renal grafts with machine perfusion equipment. Belzer and his team preserved canine kidneys for 5 days through HMP with a preservation solution that was later modified to UW solution at 5 °C [58].

Although SCS is the current gold standard of organ graft preservation due to its convenience, some animal studies have demonstrated that machine perfusion of organ grafts during ischemic storage can result in improved outcomes in comparison with SCS. In a porcine model of kidney transplantation, for example, SCS and HMP of renal grafts for 20 h followed by autotransplantation and removal of the contralateral kidney resulted in decreased proteinuria, decreased release of renal damage markers into the urine, and lower peak serum creatinine upon transplantation compared to grafts preserved by SCS [59]. Moreover, a porcine study showed that HMP of previously SCS-kept renal grafts for a few hours before their transplantation may result in comparable outcomes to continuous HMP throughout ischemia [60,61]. Advantages of HMP over SCS have also been demonstrated in clinical outcomes through a randomized controlled trial in a 2009 study [62]. This study revealed that storage of deceased donor kidneys (both DCD and DBD kidneys were part of the study) resulted in a lower risk for delayed graft function and was associated with a shorter duration of delayed graft function. Machine perfusion has been demonstrated to be a successful strategy, especially for preservation of DCD grafts, as machine-perfused DCD kidneys were demonstrated to exhibit less risk for delayed graft function upon transplantation. DBD kidneys typically can tolerate SCS in preservation solutions relatively well compared to DCD kidneys [63]. A porcine study showed that machine perfusion of DCD renal grafts during prolonged cold ischemia can lead to improved adverse post-transplant outcomes after transplantation in comparison to SCS [64]. In this study, the authors subjected donor kidneys to 1 h of warm ischemia in situ followed by 22 h of SCS or HMP with UW solution and IGL-1 solution. The kidneys were then autotransplanted, and the contralateral kidney was removed. Preservation of renal grafts in either of the storage solutions that were machine perfused resulted in less proteinuria and improved tubular integrity compared to renal grafts that were preserved by SCS [64]. Consistent results have been obtained in clinical trials where kidneys from the same donor were preserved by either SCS or HMP, and the kidneys that were preserved by HMP were associated with decreased risk of DGF (20% in the HMP group compared to 26% in the SCS group) [65]. However, the study did not find an association between acute rejection or recipient survival in relation to HMP or SCS [65]. A 2019 meta-analysis showed that HMP lowers the risk of DGF in both DCD and DBD kidney transplants, with a greater relative benefit observed in DCD grafts, highlighting its value in mitigating ischemic injury in this higher-risk donor type [66].

With the increasing number of DCD grafts being used in clinical practice, HMP may be an effective storage method for preserving renal grafts. Another experiment assessed the use of UW solution in HMP of DCD canine kidneys for 72 h at 4 °C, where the renal grafts were subjected to 75 min of warm ischemia, resulting in 86% survival after transplantation [67]. Improved outcomes of machine perfusion are seen in clinical data in the context of both DBD and DCD kidney transplantations. Based on clinical data, the use of HMP for storage of renal grafts decreases the risk of DGF by 23% compared to SCS [68].

Machine perfusion of kidneys during ischemia is thought to be therapeutic due to activation of certain protective pathways. One such pathway that is suggested is maintenance of flow within the graft microvasculature, which protects endothelial tissue due to promotion of vasoprotective phenotype through stimulation of Krüppel-like factor 2 (KF2) expression [18]. Another suggested pathway is the HMP facilitating the clearance of any pro-inflammatory factors, such as cytokines and DAMPs, as well as waste products through flushing the vasculature [18].

An additional benefit of HMP over SCS is that HMP can assess renal resistance and flow rate, which correlates with renal graft function [63,69]. Renal grafts with high renal resistance usually have increased susceptibility to primary non-function and DGF, and machine perfusion can assess flow parameters to screen the renal graft, allowing more predictability in the graft outcome [70]. However, even though perfusion parameters are important and are associated with graft outcomes, this association is not strong enough to make perfusion parameters a stand-alone criterion to determine eligibility of the organ.

Although MP is associated with improved outcomes, it is not preferred in the clinical setting worldwide, as the perfusion equipment is highly expensive, and it is not globally accessible, particularly in underdeveloped countries. However, HMP may be a more affordable option for healthcare systems in developed countries, as it may be effective in keeping the patients out of dialysis for longer periods [47]. According to a study performed by the National Institute for Health and Care Excellence in the UK, the cost of HMP per kidney was EUR 737, including the initial purchase cost, the annual maintenance cost, and the per-kidney preservation liquid/kit costs [71]. This investment may offset future expenses associated with delayed graft function (DGF) and early return to dialysis, which can amount to thousands of dollars. However, this analysis did not account for the additional costs of training healthcare personnel in the use of HMP, which could still substantially increase overall expenditure. Another study examining post-transplant hospital expenses reported that each additional inpatient day following renal transplantation cost approximately EUR 918–EUR 988 per patient, and confirmed that patients with DGF remained hospitalized longer than those without, thereby further increasing overall costs [72]. Authors of a porcine model that studied machine perfusion hypothesized that the pattern of flow during machine perfusion may have an impact on the graft health and function after transplantation [73]. In this study, the porcine renal grafts were subjected to either machine perfusion at a constant pressure of 90 mmHg or with addition of pulsatile pressure peaks (90/70 mmHg; 60/min) similar to the heart’s rhythm. The pulsatile pressure was found to improve vascular conductivity of the renal graft, creatinine clearance, sodium reabsorption, and decrease tubular injury [73].

### 3.4. Animal Models That Studied Normothermic Machine Perfusion (NMP)

Another avenue of research on organ storage conditions is through assessing different temperatures during machine perfusion. NMP is hypothesized to be protective during graft storage as it mimics the physiological state of the organ more closely than other storage methods [74]. It also provides the added benefit of allowing for functional testing of the organ, potentially allowing for improved monitoring and assessment prior to transplantation. One consideration of NMP is that the tissues are metabolically active compared to cold storage, necessitating supplementation of oxygen to the tissues. Addition of oxygen as a factor to storage is a condition that is independently evaluated by various animal studies, which will be discussed in greater detail in the next section.

Various animal models have been utilized to study feasibility of NMP. One study on pig kidneys evaluated NMP subsequent to SCS, where pig kidneys underwent 5 minutes of warm ischemia, 18 h of SCS, and 4 h of NMP, respectively [75]. This study revealed that NRP was effective at restoring tissue metabolism, although it did come with the drawback of activation of inflammatory markers during the NRP period. Adding NMP at the end of SCS was also studied in a clinical trial where DCD kidneys were either controlled statically as part of standard of care (control group) or were subjected to 1 h of NMP with a red blood cell-based perfusate at the end of a period of SCS [74]. However, this study found no change in the rate of DGF in the patients.

Many studies have evaluated only brief, end-ischemic NMP following SCS rather than prolonged NMP storage (18–24 h), largely because NMP requires continuous, expert oversight. At normothermia, the kidney’s high metabolic rate demands reliable oxygen delivery, tight control of perfusate electrolytes and acid–base balance, and strict asepsis; without this vigilance, extended runs carry increased risks of hypoxia, ionic derangements, acidosis/alkalosis, and bacterial contamination. A study utilizing clinically discarded human renal grafts attempted 24-h NMP storage through recirculating the urine that is produced by the kidney [76]. This study showed that 24 h of NMP maintained the tubular injury status of the organ as evidence of NMP being an effective storage strategy. Prolonged NMP was also studied through a 2025 clinical trial, where donor kidneys were subjected to up to 24 h of NMP prior to transplantation [77]. However, this study also found no significant difference in rates of DGF or renal function from the control group, which consisted of patients whose grafts were stored under the clinical standard, cold storage.

### 3.5. Animal Models That Studied Oxygenated Perfusion of Renal Grafts and Cell Death 

However, this approach does not enhance oxygen levels or improve hypoxia in the donor organ [78]. Emerging preclinical studies suggest that oxygenation of the preservation solution may have therapeutic effects to address the decreased oxygen consumption needs of grafts under hypothermic conditions. In a porcine model of DCD kidney transplantation, 60 min of warm ischemia followed by 22 h of machine perfusion with oxygen supplementation resulted in lower serum creatinine and decreased fibrosis at 3 months after transplantation compared to a control group without oxygen supplementation [79]. However, in grafts with extensive ischemic injury and impaired endogenous antioxidant defenses, direct oxygenation may paradoxically worsen injury by amplifying the production of reactive oxygen species (ROS), thereby exacerbating oxidative stress [78]. A fascinating prospective study involving hypothermic oxygenated machine perfusion (oxHMP) of non-transplantable kidneys from brain-dead patients showed the potential benefits of this process in organ preservation. The result of this study suggests that oxHMP has the potential to reduce macrophage and endothelial activation, tubular damage, and improve graft function [80]. The authors also suggested that this procedure is superior to traditional cold storage preservation methods. Another study found no statistical difference in 1-year graft survival when comparing kidneys from brain-dead donors subjected to oxHMP following SCS versus SCS alone [81]. However, one limitation of the study is the examination of only a brief period of oxHMP at the end of the preservation time instead of a longer period, which may have led to improved outcomes.

A method that is currently under evaluation to address the oxygen needs of organ grafts during ischemia is addition of oxygen carrier molecules to preservation solutions. Oxygen carriers that are currently evaluated through SCS and machine perfusion include cellular carriers (red blood cells (RBCs)), hemoglobin-based acellular carriers, and other acellular carriers.

Despite its efficiency in oxygenating the tissue, the use of whole blood as perfusate is not preferred, as it has been associated with vasoconstriction during ex vivo perfusion [82]. Moreover, defibrination or heparinization of the blood prior to perfusion has been ineffective [83,84]. Furthermore, pro-inflammatory factors released from leukocytes can also exert injury to the graft as well [85]. Some studies evaluated supplementation of preservation solutions with isolated RBCs to oxygenate the tissues. The use of RBCs has been shown to be effective in oxygenating renal tissue and has resulted in decreased inflammatory response. A study that evaluated use of RBCs revealed a similar extent of tissue injury between a control group and all experimental oxygen carrier groups, including RBCs (all groups oxygenated at 500 mmHg), but showed improved tissue oxygenation in the latter based on the level of lactate released to perfusate [86]. Moreover, this study revealed that addition of RBCs to storage solutions decreased the inflammatory response, as it resulted in a decrease in the levels of pro-inflammatory factors released by the renal graft. However, a disadvantage of using RBCs is the fact that RBCs undergo progressive hemolysis at low (4 °C) temperature, limiting their use at subnormothermic and normothermic temperatures, and resulting in decreased oxygen-carrying capacity and release of free hemoglobin, which has a half-life of a few hours [87]. When hemoglobin breaks apart, it releases heme, which can react with lipid peroxides or hydrogen peroxide to form ferryl myoglobin (Mb). This form of myoglobin generates harmful radicals that contribute to renal damage [87].

An alternative to RBCs is the use of acellular oxygen carriers, such as hemoglobin-based oxygen carriers (HBOCs). HBOCs contain hemoglobin molecules that were originally intended to be blood substitutes for transfusions, but their therapeutic potential on renal grafts to ameliorate ischemic insult is being evaluated [78]. Another advantage of using HBOCs instead of RBCs is that HBOCs are much smaller than RBCs and can enter the tissues through the endothelium and oxygenate the graft more efficiently in the microvasculature. Two HBOCs that are tested in preclinical and clinical settings are Hemopure and M101. Hemopure is made from bovine hemoglobin by chemical modification through cross-linking with glutaraldehyde [88]. A study from our research group showed that the use of Hemopure as a perfusate supplement during subnormothermic preservation of renal grafts results in adequate tissue oxygenation levels and less acute tubular necrosis, comparable to the use of diluted blood [89]. However, a concern with using Hemopure during HMP is that Hemopure was shown to be a scavenger of nitric oxide (NO) in the tissues, leading to vasoconstriction [90]. This may translate into a decrease in flow rate and an increase in renal resistance upon reperfusion of the graft, though a dedicated study has not been performed.

Another HBOC that is being investigated experimentally and clinically is M101. M101 is composed of hemoglobin obtained from a marine lugworm, *Arenicola marina*. This hemoglobin is a large complex that can bind up to 156 oxygen molecules [91]. Moreover, M101 releases oxygen against a gradient, preventing oxidative stress due to rapid oxygenation of the tissue. It is also active at a range of temperatures (4–37 °C), allowing its use at hypothermic, subnormothermic, and normothermic conditions. M101 has a half-life of 300 and 3000 h in HTK and UW solutions, respectively, allowing for its use for storage of renal grafts for prolonged periods of time (18–24 h) [91]. In a porcine model of kidney transplantation, supplementation HTK and UW solutions with M101 during 24 h of SCS and upon a 3-month monitoring period after transplantation resulted in M101-stimulated recovery of renal graft function, improved creatinine excretion, and preserved renal architecture with less inflammation, proteinuria, and fibrosis compared to control groups stored in HTK and UW solutions without M101 supplementation [91]. Moreover, M101 may also have benefits for storage of DCD kidneys. In a porcine model of DCD kidney transplantation, for example, addition of M101 to preservation solution during 23 h of SCS and HMP of DCD renal grafts at 4 °C after 1 h of WIT resulted in improved short- and long-term graft function at 1 and 3 months after transplantation, with significantly less fibrosis compared to control and SCS groups [92]. One significance of these porcine studies is that they involved SCS, the current clinical gold standard for organ preservation. This suggests that M101 may be beneficial in systems that do not have access to perfusion equipment. Also, one advantage of the use of M101 in HMP is that it replaces the oxygenating pump that functions to constantly supply the perfusate with oxygen. This makes the use of HMP less expensive compared to the use of an oxygenating pump. In a similar porcine model of DCD kidney transplantation, HMP of renal grafts with M101 supplementation in the presence or absence of continuous oxygenation improved graft quality and function and reduced post-transplant complications [93]. This observation implies that the use of M101 does not require constant oxygen supplementation to have therapeutic effects during machine perfusion of organ grafts. It is important to note that part of the therapeutic effects of M101 is attributable to its superoxide dismutase activity, which reduces production and deleterious effects of ROS [91]. Taking advantage of the gradual oxygen delivery to the tissues, another study evaluated whether supplementation of M101 to static preservation solution during SCS in an in vivo porcine model of transplantation, where kidneys underwent 24 h of SCS prior to autotransplantation with removal of the contralateral kidney [94]. This study suggested that kidneys preserved in M101 in static storage had improved function recovery, preservation of integrity, and reduced inflammation. The results from this study are important as the authors demonstrated that M101 addition can improve transplantation outcomes without the need for costly HMP equipment. Although multiple studies have demonstrated the efficacy of M101, most have been conducted in Europe. Trials in other regions are still needed to determine whether M101 offers similar benefits under different healthcare systems and regulatory frameworks, as well as with varying preservation and handling practices. Table 4 summarizes various storage techniques discussed throughout this review.

## 4. Animal Models to Study Therapeutics for Deceased Donor Kidneys

Preservation solutions such as the UW solution that are commonly used for storage of renal grafts are formulated to contain various reagents and pharmacological supplements that address pathological mechanisms during ischemia and reperfusion of the graft. However, new areas of research investigate supplementation of these storage solutions with further ingredients to ameliorate this damage to a greater extent.

### 4.1. Animal Models That Studied Supplements to Storage Solutions of Deceased Donor Kidneys

ROS generation is a major part of damage due to renal IRI, as ROS leads to structural and DNA damage, production of toxic metabolites, and activation of cell death pathways in the cells. Thus, some preclinical studies focused on supplementation of the preservation solutions with antioxidant compounds to counteract the effects of ROS on cellular compartments. One antioxidant that is evaluated for its effects against IRI is vitamin C. Vitamin C is a water-soluble compound with intracellular and extracellular antioxidant properties at micromolar concentrations [95]. It can directly react with ROS by acting as a proton or electron donor through its lactone ring, protecting cellular structures against oxidative damage. Vitamin C has been shown to inhibit lipid peroxidation under conditions of increased oxidative stress in vivo [96], potentially resulting in decreased production of the cytotoxic compounds MDA and 4-HNE. In addition, vitamin C has also been suggested to improve DNA repair processes in response to oxidative stress due to gamma irradiation [97]. To investigate the therapeutic effects of vitamin C supplementation on transplant organs, Ostróżka-Cieślik et al. [88] evaluated effects of vitamin C supplementation to preservation solution in a porcine model of kidney transplantation. The porcine kidneys were nephrectomized and were perfused with either Biolasol solution alone or with vitamin C supplementation for 48 h. Interestingly, vitamin C-treated renal grafts maintained more cytoskeletal integrity than control grafts without vitamin C supplementation [88]. In an in vitro model of perfused porcine kidneys, graft preservation in Ringer’s solution supplemented with vitamin C markedly decreased oxidative stress relative to a control group, but did not significantly influence the extent of damage markers released and the extent of damage [98,99].

Quercetin is another antioxidant that is under experimental investigation in the context of organ transplantation. Besides its antioxidant property, quercetin possesses anti-inflammatory and anti-apoptotic properties, all of which partly contributed to improved graft quality and function upon addition to the UW solution [100]. However, quercetin is also an inhibitor of heat shock proteins (HSPs), and increased HSP activity has been shown to be therapeutic against IRI [101]. Apart from these antioxidants, inhibitors of matrix metalloproteinases (MMPs; enzymes that function to remodel the extracellular matrix) have also been recently identified to improve graft function when added to organ preservation solutions. In a rat model of cold ischemic perfusion, supplementation of the perfusion solution with DOXY, an inhibitor of MMPs, provided graft protection by significantly decreasing the level of damage markers released into the perfusion solution [102].

Alpha-lipoic acid (ALA) is another antioxidant compound that has been shown to alleviate damage due to IRI. ALA is endogenously produced in the mitochondria from octanoic acid and is known to neutralize ROS by directly reacting with them [103]. As an FDA-approved drug for the treatment of diabetic neuropathy, ALA has been shown to have anti-inflammatory properties, inhibiting nuclear translocation of NF-кB, which is partially responsible for its therapeutic effects against IRI [104]. In a rat model of renal IRI, induction of 40 min of warm ischemia through clamping of the renal pedicles showed that administration of ALA to rats 24 and 48 h before induction of warm ischemia and 6 and 24 h after warm ischemia resulted in improved creatinine clearance and lower plasma creatinine 2 days after warm ischemia [105].

Another compound that is currently studied as a pharmacological supplement to storage solutions is hydrogen sulfide (H_2_S). H_2_S is the third established member of a family of gaseous signaling molecules that is endogenously produced in all mammalian cells using L-cysteine and 3-mercaptopruvate through the activity of the three enzymes, cystathionine β-synthase (CBS), cystathionine γ-lyase (CSE), and 3-mercaptopyruvate sulfurtransferase (3-MST) [106]. Besides inhalation as the classical form of administration, H_2_S can also be administered through donor compounds such as sodium sulfide (Na_2_S), sodium hydrosulfide (NaHS), GYY4137, AP39, garlic-derived polysulfides, and sodium thiosulfate (STS; clinically viable). At high concentrations of 500–1000 ppm, H_2_S can cause death by reversibly inhibiting complex IV of the electron transport chain [107]. However, H_2_S has been shown to have therapeutic properties at low physiological concentrations [108]. Exogenous administration of H_2_S to mice has been shown to induce a reversible hypometabolic state characterized by a decrease in oxygen consumption, carbon dioxide output, and core body temperature. Moreover, pretreatment of mice with gaseous H_2_S prior to subjecting them to low (5%) oxygen conditions allowed survival for 6 h in this hypoxic environment compared to untreated mice that could not exceed 20 min [109]. This effect was attributed to the reversible inhibitory effect of H_2_S on the mitochondrial electron transport chain, resulting in a lower rate of oxidative phosphorylation and a decreased oxygen demand [109].

Effects of H_2_S on the rate of metabolism through its effect on mitochondria led scientists to study H_2_S as a therapeutic against IRI of organs, with the hypothesis of reducing their oxygen demand during the ischemic period, ultimately leading to downregulation of cell death pathways. However, the therapeutic effects of H_2_S under hypoxic conditions are not limited to its effects on metabolic rate. In a mouse model of myocardial IRI, administration of low concentrations of H_2_S did not influence the metabolic rate of mice but still had some protection against cell death and granulocyte infiltration caused by myocardial IRI compared to the untreated control group [110]. Interestingly, a superior protection was observed in the group that received a higher dose and had a reduced metabolic rate [110]. In addition to its inhibitory activity on oxidative phosphorylation, H_2_S can regulate functions of various proteins that are part of several molecular pathways, including apoptotic, inflammatory, proliferative, and metabolic pathways [106]. Moreover, evidence from isolated mouse mitochondria suggests that H_2_S may also be protective against apoptosis and oxidative stress-induced necrosis by inhibiting mPTP opening in high Ca^2+^ conditions [111]. H_2_S also has antioxidant properties against ROS as it stimulates production of glutathione (GSH), the most abundant naturally occurring antioxidant, to directly scavenge ROS [112]. As a gasotransmitter, H_2_S has also been shown to have vasodilatory effects on the endothelium [113].

Our research group was the first to provide experimental evidence that H_2_S donors such as NaHS, GYY4137, AP39, and STS can be used as pharmacological supplements to preservation solutions in the context of solid organ transplantation, which typically involves extensive cold ischemia during graft preservation [114]. Using rodent and porcine models of kidney transplantation, our research team demonstrated the therapeutic effect of supplementing organ preservation solutions at different preservation temperatures and varying preservation duration with H_2_S donor compounds by safely extending cold ischemic time with limited tissue injury, and improving renal graft quality and function and post-transplant adverse outcomes [113,114,115,116,117]. Table 5 is a summary of the therapeutics used to mitigate renal IRI in various experimental models.

### 4.2. Animal Studies That Evaluated Donor Strategies

Although the majority of studies have focused on storage conditions of organ grafts, accumulating evidence suggests that interventions applied to the organ donor prior to procurement can confer additional protection. If organ procurement occurs in a controlled manner—where the time of death can be determined based on withdrawal of life support from the donor (such as in Category III DCD or DBD donation)—such donor-level therapeutics may be applied to improve renal graft preservation [34].

One donor technique evaluated extensively in animal studies is ischemic preconditioning (IPC). IPC involves exposing tissue to short periods of ischemia (5–15 min) before a prolonged ischemic insult, which increases tolerance against subsequent ischemic damage [118]. In a murine model, IPC kidneys underwent 15 min of warm ischemia followed by 10 min of reperfusion before 3 h of cold storage and transplantation. Compared to control grafts, IPC-treated kidneys exhibited significantly reduced tubular injury and lower TNF-α expression 24 h post-transplantation [118]. While these results are encouraging, the clinical feasibility of IPC is limited, as it requires direct manipulation of the donor organ or donor circulation prior to procurement, which may not be ethically or practically possible in most clinical settings. Invasive procedures carry a risk of donor instability and are, therefore, rarely implemented outside controlled experimental conditions.

To overcome these challenges, remote ischemic conditioning (rIPC) has emerged as a more feasible alternative. rIPC consists of inducing transient ischemia in a distant tissue (e.g., a limb) through cycles of occlusion and reperfusion, typically using a blood pressure cuff. In murine models, rIPC applied to the limb resulted in reduced plasma creatinine and urea, increased creatinine clearance, and marked downregulation of renal KIM-1, with multiple short cycles of rIPC showing superior protection [119]. rIPC has also demonstrated systemic protective effects, conferring benefits beyond the kidney that could be advantageous when multiple organs are procured for transplantation. Importantly, rIPC is clinically attractive because it is minimally invasive, requires no surgical intervention or specialized equipment, and can be applied rapidly and safely at the bedside.

The clinical relevance of rIPC has been supported by large animal studies. In a porcine model of DBD kidney transplantation, recipient rIPC prior to reperfusion improved cortical and medullary plasma perfusion, enhanced early single kidney GFR, and stabilized mean arterial pressure, which collectively led to better post-transplant outcomes compared with controls [120]. These findings suggest that rIPC may reduce the incidence of delayed graft function (DGF) and potentially decrease the need for acute dialysis after transplantation.

Another donor-level therapeutic approach is the administration of protective compounds prior to procurement. For instance, administration of N-octanoyl dopamine (NOD), a synthetic dopamine derivative, in a rat model of DBD kidney transplantation improved GFR, reduced immune-mediated injury, and attenuated inflammation without hemodynamic side effects [121]. Such pharmacological preconditioning strategies highlight the potential of donor-targeted interventions, though their safety and efficacy remain to be validated in clinical trials.

## 5. Considerations and Limitations of Animal Models of Kidney Transplantation

Although animal models are important tools for testing new methods for renal graft preservation and transplantation, findings from them do not always translate to humans. Rodents are inexpensive and can produce quick results. However, anatomical differences between human and rodent urinary systems (e.g., unilobular and unipapillary kidneys in rodents vs. multilobular and multipapillary in humans) set a limitation on the usefulness of rodent studies in the context of kidney transplantation. In addition, the upper limit of WIT in rat models of DCD kidneys is determined to be 40 min to allow for functional recovery in the donor kidney, whereas that in humans ranges from 30 to 120 min [115]. The difference in WIT between donor kidneys of rats and humans is significant enough to introduce significant variation in the degree of ischemic injury. Canine models were very common in the early studies of kidney transplantation. However, an important limitation of canine IRI models is that canine kidneys are relatively resistant to IRI compared to human kidneys [59]. This explains why canine kidneys can be preserved for up to 3–5 days. Considering these limitations, porcine models are preferred due to similarity to human kidneys in terms of genetics, embryological development, and morphological features, and responses to injury. Nonetheless, it is important to note that numerous interventions highly effective in animal models have failed to show consistent benefit in clinical trials of kidney transplantation. For example, rIPC—the application of brief, controlled episodes of ischemia to a distant organ or limb to induce systemic protection—has demonstrated robust protective effects against renal ischemia in both rat and porcine models, as discussed in the previous section [120,122]. However, a recent systematic review and meta-analysis of randomized controlled trials in human kidney transplantation found that rIPC provided no significant reduction in mortality, delayed graft function, or rejection rates, highlighting the persistent gap between animal success and clinical outcomes [123]. Another example of a method that was effective on animal models but not in the clinic was discussed in a previous section, where therapeutic effects of NMP after a period of static storage were not replicated in the clinic through a clinical trial. This discrepancy highlights the macro-level translational gap between preclinical and clinical research: while animal studies provide mechanistic insights and proof-of-concept evidence, the complexity and heterogeneity of human donors and recipients often limit direct applicability. Therefore, while porcine models remain the most clinically relevant, careful interpretation of animal data and robust human trials remain essential for evaluating the true efficacy of novel preservation strategies such as machine perfusion.

## 6. Conclusions

This review has highlighted the advancements and challenges in deceased donor kidney transplantation. We examined various underlying mechanisms and processes implicated in organ damage following kidney transplantation, including IRI, oxidative stress, and inflammatory responses. The examination of different types of deceased donor kidneys, including DBD, DCD, and DBCD, provided insights into the unique and similar challenges encountered for each type. Most notably, our review investigated animal models of static cold storage, machine perfusion, possible supplementation of preservation solutions with therapeutics, and donor preconditioning strategies. This information not only adds to our understanding of kidney transplantation but also provides insights into potential therapeutic avenues that could be further explored in animal studies and in clinical settings. In conclusion, deceased kidney transplantation remains a complex and nuanced field with a lot of promise. Through more research and building on our knowledge of the underlying mechanisms of renal damage, along with potential therapeutic strategies, we can strive for improved outcomes of organ transplantation with improved quality of life for patients living with ESRD.

## Figures and Tables

**Table 1:** Maastricht classification of DCD organs based on circumstances of death. From Park et al. [35]. Globally, Class III is the most commonly used category of DCD donors. Classes I and II are rarely utilized because they are associated with prolonged and often uncertain warm ischemia times, resulting in unpredictable recipient outcomes. Classes IV and V are still employed in clinical practice but occur far less frequently than Class III.

Category	Definition	Type of DCD
I	Dead when arrived at hospital (1) Cardiocirculatory death outside hospital with no witnesses. (2) Cardiocirculatory death outside hospital with witnesses or rapid resuscitation attempt.	Uncontrolled
II	Unsuccessful cardiopulmonary resuscitation: witnessed cardiac arrest outside the hospital with unsuccessful cardiopulmonary resuscitation	Uncontrolled
III	Cardiac arrest following the withdrawal of life-sustaining treatments but not considered to be brain dead	Controlled
IV	Cardiac arrest in the process of the determination of death by neurological criteria after brain death or after such determination has been performed, but before being transferred to an operating room	Uncontrolled
V	Cardiac arrest in hospital patients	Uncontrolled

**Table 2:** Summary of outcomes based on deceased donor type.

Rate of Outcomes	Type of Deceased Donor Graft	
	DCD	DBD
Discard Rates	34% [41]	24% [41]
DGF	30–50% [31]	20% [42]
5-Year Survival	76% [31]	75% [36]

**Table 3:** A summary of the graft storage solutions and their use in experimental models.

Solution	Key Composition/Innovations	Experimental Model	Preservation Duration (0–4 °C)	Main Findings/Outcomes
Hank’s Solution (1963)	Mimicked extracellular fluid	Canine kidneys	Up to 12 h	Kidneys functioned immediately post-transplant with normal creatinine. Failed at 24 h due to cold ischemia injury [46].
Saline	Simple isotonic saline	Canine kidneys	Up to 16 h	Kidneys resisted ischemia up to 16 h at 0–4 °C [49].
Solution C	Simulated intracellular fluid (↑ K^+^, ↓ Na^+^) to reduce ischemia-induced edema	Canine kidneys	Up to 30 h	Lower serum creatinine after prolonged ischemia. Ineffective if >20 min warm ischemia before storage [49,50].
Euro-Collins Solution	Modified Collins: ↑ Glucose (195 vs. 140 mmol), removed Mg^2+^	DCD canine model (35 min warm ischemia)	Up to 24 h	Effectively preserved renal grafts; improved post-transplant outcomes. Adopted clinically in Europe (1980s) [51].
University of Wisconsin (UW) Solution	Intracellular-type solution with impermeants (lactobionate, raffinose), antioxidants (glutathione), colloid (HES)	Canine kidneys	Up to 72 h	Significantly higher graft viability and function after transplantation compared to EC solution [52,54].

↑ = Increase; ↓ = Decrease.

**Table 4:** Comparison of advantages and disadvantages of different methods of organ storage.

Storage Method/Reagent	Advantages	Disadvantages
Static Cold Storage (SCS)	Accessible Affordable Minimal expertise needed	Increased risk of DGF, especially in DCD kidneys
Hypothermic Machine Perfusion (HMP)	Improved outcomes of DGF (particularly for DCD kidneys)	Costly Requires expertise to operate
Normothermic Machine Perfusion (NMP)	Allows for closer monitoring of graft function.	Costly Requires expertise Was not shown to be particularly therapeutic through multiple clinical studies [74,77]
Oxygenated HMP (oxHMP)	Provides some oxygen during hypoxia to prevent some ischemic damage [79]	More costly than HMP Inefficient oxygen delivery into the tissue May be more damaging to extensively injured grafts due to accelerating their metabolic rate [78]
Whole Blood Perfusion	More efficient in oxygenation than oxHMP [82]	Hard/inconvenient to source blood Whole blood has vasoconstrictive properties [82] Risk of clot formation inside the kidney [82,83,84] Pro-inflammatory molecules released from leukocytes can cause further inflammation in the kidney [85]
Perfusion with Isolated Red Blood Cells (RBCs)	Efficient in oxygenating the tissue without inflammatory contribution from the leukocytes [86] Shown to be anti-inflammatory	Hard/inconvenient to source blood to isolate the RBCs from. RBCs are prone to hemolysis at hypothermia, leading to free hemoglobin ultimately dissociating to release the toxic heme molecule [87]
Acellular Oxygen Carriers (M101, Hemopure)	More consistent/reliable to source compared to blood. Efficiently oxygenates the tissue [89] Wide range of effective temperatures [91] Some reagents (M101) release oxygen in a gradient, ensuring constant and gradual oxygen supplementation to the graft [91]	More costly than regular HMP and oxHMP Not approved globally by different health regulations.

**Table 5:** Summary of compounds used in preclinical models to mitigate renal IRI.

**Compound**	**Mechanism of Action**	**Experimental Model**	**Main Findings/Outcomes**	**Notes/Limitations**
Vitamin C	Water-soluble antioxidant; donates protons/electrons via lactone ring; inhibits lipid peroxidation (↓ MDA, 4-HNE); enhances DNA repair	Porcine kidney perfusion with Biolasol ± Vit C [88]; in vitro porcine kidneys perfused in Ringer’s ± Vit C [99]	Preserved cytoskeletal integrity vs. control; ↓ oxidative stress in vitro	Did not significantly reduce release of injury markers or overall tissue damage
Quercetin	Antioxidant, anti-inflammatory, anti-apoptotic; improves graft quality/function	UW solution + quercetin [100]	Improved graft function with supplementation	Also inhibits heat shock proteins (HSPs), which may counteract protection since ↑HSPs are beneficial in IRI [101]
Doxycycline (DOXY)	MMP inhibitor; prevents ECM degradation and inflammation	Rat model of cold ischemic perfusion [102]	↓ Damage markers released into perfusate; improved graft protection	Limited to preclinical data
Alpha-Lipoic Acid (ALA)	Endogenous mitochondrial antioxidant; directly scavenges ROS; inhibits NF-κB translocation (anti-inflammatory)	Rat renal IRI model (40 min warm ischemia; ALA given 24/48 h before and 6/24 h after ischemia) [105]	↑ Creatinine clearance; ↓ plasma creatinine at 2 days post-IRI	Already FDA-approved for diabetic neuropathy [104]
Hydrogen Sulfide (H_2_S) & Donors (NaHS, Na_2_S, GYY4137, AP39, STS, garlic polysulfides)	Modulates mitochondrial respiration (↓ O_2_ consumption, reversible inhibition of complex IV); induces hypometabolic state; antioxidant via ↑ GSH; inhibits mPTP opening; vasodilatory	Rodent and porcine renal transplant models [113,114,115,116,117]; myocardial IRI mouse models [110]	Safely extended cold ischemic time; ↓ tissue injury; improved graft quality and post-transplant outcomes	Toxic at high concentrations (500–1000 ppm); dose-dependent; STS is clinically viable donor

↑ = Increase; ↓ = Decrease.

## Data Availability

No new data were created or analyzed in this study. Data sharing is not applicable to this article.
